# Congenital Laryngeal Cyst: A Rare Cause of Polyhydramnios

**Published:** 2013-05-02

**Authors:** Hatice Tatar Aksoy, Nilda Süslü, Gamze Demirel, İstemihan Çelik, Fuat Emre Canpolat, Ömer Erdeve, Umut Akyol, Ugur Dilmen

**Affiliations:** Division of Neonatology, Zekai Tahir Burak Maternity Teaching Hospital, Ankara, Turkey.; Division of Neonatology, Department of Pediatrics, Ankara University School of Medicine Children’s Hospital, Ankara, Turkey.; Division of Neonatology, Zekai Tahir Burak Maternity Teaching Hospital, Ankara, Turkey.; Division of Neonatology, Zekai Tahir Burak Maternity Teaching Hospital, Ankara, Turkey.; Division of Neonatology, Zekai Tahir Burak Maternity Teaching Hospital, Ankara, Turkey.; Division of Neonatology, Department of Pediatrics, Ankara University School of Medicine Children’s Hospital, Ankara, Turkey.; Department of Otorhinolaryngology, Hacettepe University Faculty of Medicine, Ankara, Turkey.; Division of Neonatology, Zekai Tahir Burak Maternity Teaching Hospital, Ankara, Turkey, and Department of Pediatrics, Yildirim Beyazit University School of Medicine, Ankara, Turkey.

**Keywords:** Laryngeal cyst, Polyhydramnios, Respiratory distress

## Abstract

Congenital laryngeal cyst is a rare cause of airway obstruction that may require urgent diagnosis and treatment. We report a case of a neonate having history of polyhydramnios and severe respiratory distress at birth. A laryngeal cyst detected during intubation. The outcome of laryngoscopic treatment of the cyst was favorable.

## INTRODUCTION

Benign congenital laryngeal cysts are rare entities with an estimated incidence of <2 per 100,000 live births [1]. They can cause severe respiratory distress immediately after birth [2]. The etiology is not clear. With improvement in fetal imaging modalities, laryngeal cysts can be diagnosed antenatally in the second trimester [3], which helps in planning immediate perinatal management. Undiagnosed cases of laryngeal cyst are associated with high mortality (40%), thus underscoring the importance of antenatal diagnosis. We present a case of newborn with laryngeal cyst detected during intubation who received successful treatment with microlaryngoscopic excision and cauterization.

## CASE REPORT

A 2900 g, term male newborn, with history of maternal polyhydramnios was admitted to the NICU because of severe cyanosis and respiratory insufficiency that developed immediately after birth. During intubation, a large laryngeal mass was noted. Laryngotracheobronchoscopy (LTB) under general anesthesia revealed a cystic mass in the left supraglottic region of the larynx (Fig. 1). The cyst was punctured and the mucoid fluid drained by suction to prevent aspiration. In the next few days after extubation, progressive inspiratory stridor developed. A second LTB showed a large recurrent cyst. Microlaryngoscopic excision of the prominent part of the cyst was performed (Fig. 2). Pathologic examination was consistent with a laryngeal cyst. For one week, the neonate could not be extubated. Consequently, peroral corticosteroid therapy and inhalation therapy with salbutamol and ipratropium bromide were administered. After a few days, the neonate was extubated and discharged well on day 30 of life. 

**Figure:**
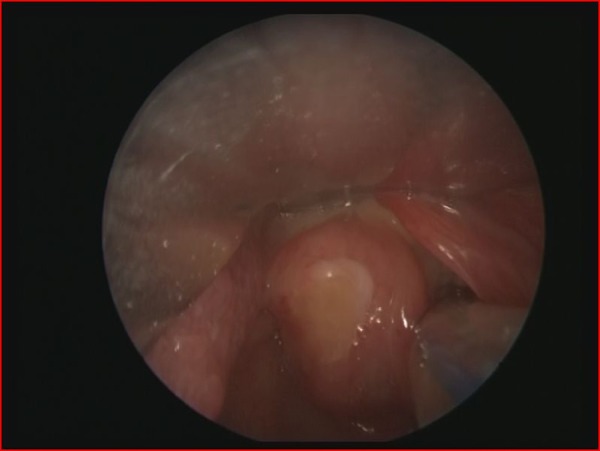
Figure 1: Laryngoscopic appearance of laryngeal cyst

**Figure:**
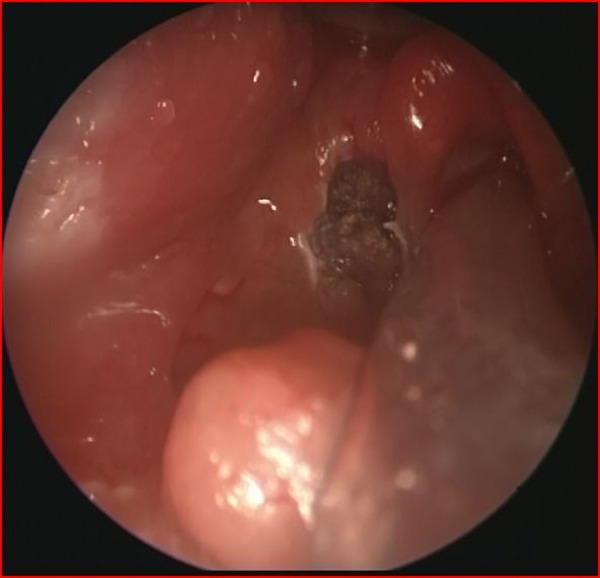
Figure 2: Laryngoscopic appearance of cauterized cyst

## DISCUSSION

Congenital laryngeal cysts are rare but may be fatal [4]. Apart from acute life threatening airway obstruction, presenting symptoms include stridor, hoarse cry, apnea, cyanosis, chest retractions, feeding problems and failure to thrive. Severe antenatal obstruction may lead to polyhydramnios and pulmonary hypoplasia. Compression of the trachea, cervical vessels and hypoglossal nerve have also been reported [1]. Antenatally, the diagnosis can be suspected on ultrasonography, which can show either the cyst or polyhydramnios [5]. In our case, polyhydramnios was detected prenatally but laryngeal cyst was not detected. Fortunately, it was detected during intubation performed for respiratory insufficiency. 

Polyhydramnios complicates 4% of pregnancies [6]. Polyhydramnios is idiopathic in approximately 35% to 65% of cases. However, with advancement in technology, the cause can be detected in most of the cases. In case of polyhydramnios, the pharynx and neck should be investigated thoroughly in order to identify lesions that could cause neonatal ventilation problems. About 18% of cervical congenital abnormalities can lead to polyhydramnios [7]. Congenital laryngeal cysts are rare, but are a possible cause of early neonatal respiratory distress. Due to their size and position, they can cause total or subtotal airway obstruction with consequent respiratory insufficiency and require emergency endotracheal intubation as happened in the index case [8]. 

Surgical procedures that are proposed for the treatment of congenital laryngeal cysts vary: While smaller lesions can be excised, marsupialised during endoscopy, larger or recurrent cysts may require a lateral cervical approach. In our patient, laryngoscopic drainage, excision of the prominent part and cauterization were performed. Microlaryngoscopic excision in our case assisted with endoscopic cauterization was safe and effective, with minimal morbidity. 

To conclude, in a neonate with respiratory insufficiency and a prenatal history of polyhydramnios, one should always consider the possibility of congenital laryngeal cysts and be alert that a careful observation during intubation can lead to diagnosis and prompt life saving management.

## Footnotes

**Source of Support:** Nil

**Conflict of Interest:** None declared

